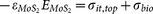# Two-dimensional Layered MoS_2_ Biosensors Enable Highly Sensitive Detection of Biomolecules

**DOI:** 10.1038/srep07352

**Published:** 2014-12-17

**Authors:** Joonhyung Lee, Piyush Dak, Yeonsung Lee, Heekyeong Park, Woong Choi, Muhammad A. Alam, Sunkook Kim

**Affiliations:** 1 Multi-Functional Nano/Bio Electronics Lab., Department of Electronics and Radio Engineering, Kyung Hee University, Gyeonggi 446–701, South Korea; 2 School of Electrical and Computer Engineering, Purdue University, West Lafayette, IN 47907, USA; 3 School of Advanced Materials Engineering, Kookmin University, Seoul 136–702, Korea

## Abstract

We present a MoS_2_ biosensor to electrically detect prostate specific antigen (PSA) in a highly sensitive and label-free manner. Unlike previous MoS_2_-FET-based biosensors, the device configuration of our biosensors does not require a dielectric layer such as HfO_2_ due to the hydrophobicity of MoS_2_. Such an oxide-free operation improves sensitivity and simplifies sensor design. For a quantitative and selective detection of PSA antigen, anti-PSA antibody was immobilized on the sensor surface. Then, introduction of PSA antigen, into the anti-PSA immobilized sensor surface resulted in a lable-free immunoassary format. Measured off-state current of the device showed a significant decrease as the applied PSA concentration was increased. The minimum detectable concentration of PSA is 1 pg/mL, which is several orders of magnitude below the clinical cut-off level of ~4 ng/mL. In addition, we also provide a systematic theoretical analysis of the sensor platform – including the charge state of protein at the specific pH level, and self-consistent channel transport. Taken together, the experimental demonstration and the theoretical framework provide a comprehensive description of the performance potential of dielectric-free MoS_2_-based biosensor technology.

Highly sensitive and rapid detection of biomolecules is essential for biosensors used in clinical, military, or environmental applications. Among various biosensing platforms, biosensors based on field effect transistors (FETs) have been widely investigated to detect a variety of target analytes due to their high sensitivity, label-free detection capability, and compatibility with commercial planar processes for large-scale circuitry^1^,^2^,^3^. Especially, the integration of nanomaterials, such as Si-nanowire (NW), ZnO nanowire, single-walled carbon nanotube (SWNT), or graphene, in a FET configuration offers significant advantages over the label-based techniques for the detection of biological analytes^4^,^5^,^6^. FET biosensors have been demonstrated to be effective in recognizing binding events of charged or polar biological species, because the electrostatic interaction between biomolecules and gate dielectric or channel can give rise to conductance modulation in transistors^7^.

The biosensors based on one-dimensional (1D) NWs and SWNTs are highly sensitive, but prone to a large deviation of device-to-device performance due to the uncontrolled variations in thickness, purity, chirality, and crystal defects. Additional challenges include the lack of reliable processes of integrating 1D nanomaterials into transistors. On the other hand, the classical Si-FET sensors are easily integrated into massively parallel platform; however, the sensor must be protected from the salt solution by insulators^7^. As a result, the sensitivity is reduced on two counts: the coupling of the biomolecule to the channel is compromised, the mobility of electrons in the channel is degraded due to surface roughness scattering, and the traps in the oxide increase 1/f noise^8^,^9^. Most importantly, the hydrophilic nature of the oxide surface makes surface functionalization difficult and the binding event less efficient.

A new generation of two-dimensional (2D) nanomaterials, such as graphene or transition metal dichalcogenide (TMD), might provide an opportunity for an ultra-sensitive biosensor application because they are compatible with commercial planar processes for the large-scale circuits^10^,^11^,^12^,^13^. While the zero bandgap of graphene limits the sensitivity of graphene FET-based biosensors, the existence of bandgap in TMDs could enable highly sensitive detection of biomolecular targets by TMD FET-based biosensors^14^. Interestingly, recent reports exhibit that the surrounding net-charges can easily bring the variation of carrier transport in 2D crystals^15^,^16^,^17^. Such highly sensitive electrical properties of 2D layered semiconductors are attractive for biosensors since the binding event at the interface between MoS_2_ and charged biomolecules can be monitored by a direct change of the transistor performance metrics including threshold voltage (V_t_), field-effect mobility (μ_eff_), and subthreshold swing (SS). The variation of V_t_ or the conductance for a transistor can be also utilized to measure the number of charged biomolecules onto MoS_2_ crystals quantitatively. Furthermore, the application of MoS_2_ FET-based biosensors can become even more promising due to the recent progress in large-area synthesis of 2D MoS_2_ using chemical vapor deposition (CVD) methods^18^,^19^.

Recently, Sarkar *et al.*^14^ have reported detection of streptadavin using MoS_2_ biosensor with HfO_2_ gate dielectric functionalized with biotin. As discussed previously, many gate dielectrics including HfO_2_ are hydrophilic and have relatively low affinity to biomolecule adsorption. Therefore, in order to monitor the binding events, the oxide surface needs to be treated with additional chemicals, such as APTES (3-aminoproplytriethoysilane)^20^. Most of the sensing experiments take place in ionic media. Treatment with chemicals introduces an extra layer of molecules which further increases the separation between the charged biomolecule layer from the sensor surface. This can considerably deteriorate the device sensitivity due to ionic screening^21^. Recently, Gaur *et al.*^22^ have shown that crystalline MoS_2_ deposited on oxide surface has hydrophobic nature and hence it is expected to have a higher affinity to biomolecule binding. Therefore, MoS_2_ can serve the dual purpose of surface-adsorption layer as well as sensing layer. This remarkable prospect of oxide-free operation of MoS_2_ biosensors has not been extensively explored in the literature.

The present work demonstrates the implementation of MoS_2_ biosensors to electrically detect prostate specific antigen (PSA) in a highly sensitive and label-free manner without the need of a chemically treated gate dielectric. The device configuration of our MoS_2_ biosensors is utilized as bottom-gated MoS_2_ FETs with higher sensitivity as well as simpler device structure for oxide-free operation. Here, the nature of hydrophobic MoS_2_ surface (the contact angle ~75.77°) allows physical adsorption of biomolecules to the sensor surface. Moreover, the use of off-current as an indicator of sensitivity, instead of the subthreshold swing or shift in the threshold voltage, allows additional improvement in device sensitivity in our MoS_2_ FET biosensors. Theoretical calculation of the MoS_2_ biosensor operation provides a systematic understanding of the experimental results of the MoS_2_ biosensors. Theoretical analysis also predicts that controlling defect density and interface trap density is necessary to additionally improve the device sensitivity.

Prostate specific antigen (PSA) has been identified as one of the reliable clinical tools for diagnosing and monitoring prostate cancer. Therefore, the accurate and sensitive detection of PSA at the earliest stage is important for prostate cancer diagnostics and treatment. PSA is typically a negatively charged molecule. To explore the potential for quantitative detection of PSA target with 2D multilayer biosensor, we fabricate bottom-gated MoS_2_ FETs with higher sensitivity with simpler device structure than those in literature. Fig. 1(a) illustrates the schematic architecture to show label-free immunoassay between an anti-PSA modified MoS_2_ nanosheet and PSA target in non-aqueous media, unlike the conventional micro-fluidic bio-chip. Anti-PSA (antibody) is physisorbed nonspecifically on the sensing area, and then PSA (antigen) is selectively bound to the immobilized antibody on MoS_2_ nanosheet, resulting in a classic label-free immunoassay format. It is well known that the proteins retain their ionization state corresponding to the pH of the aqueous solution (PBS) from which they were lyophilized (called “pH memory”)^23^,^24^,^25^. Therefore, the surface charge due to lyophilized proteins results in a stable change in the conductance of the transistor without the need of reference electrode, which is reflected in the drain current as will be discussed later. The first step of fabricating transistors is mechanical exfoliation from bulk MoS_2_ (SPI Supplies, USA) using scotch tape. Detached thin-film MoS_2_ flakes are transferred on highly p-doped Si substrate with SiO_2_ (300 nm). Atomic force microscopy (AFM) measurement shows the thickness of MoS_2_ flakes in the range of 20–80 nm. Then silicon wafers with SiO_2_ and MoS_2_ are cleaned in acetone and IPA for 1 h in order to remove residues. Arrays of 200 × 200 μm^2^ contacts are patterned by conventional photolithography and lift-off process. Metal contacts of Ti/Au (20 nm/300 nm) are subsequently deposited by e-beam evaporation. To reduce contact resistance, samples are annealed at 200°C with 100 sccm of Ar gas and 10 sccm of H_2_ gas in a thermal vacuum tube.

As mentioned previously, the hydrophobicity of the MoS_2_ surface is a key enabling feature, since hydrophobic surfaces have been well known to yield higher affinity of protein-surface adsorption than hydrophilic surfaces. In order to determine degree of hydrophobicity of MoS_2_, we measure contact angles of MoS_2_, Au, and SiO_2_ surface to explore the potential of protein adsorption on MoS_2_ without a specific surface treatment. In Fig. 1(b), the contact angle of MoS_2_, Au, and SiO_2_ are observed as 75.77°, 75.72°, and 23.1°, respectively. While SiO_2_ surface is hydrophilic with a contact angle of 23.1°, MoS_2_ and Au surfaces are relatively hydrophobic with contact angles of ~75°. Due to the extremely low contact angles, SiO_2_ is expected to yield low affinity of protein-surface adsorption, and require an additional treatment of APTES coupled with oxygen plasma-cleaned Si-surface to obtain terminal amine groups^26^. Since antibody as well as protein is easily immobilized without the complicated surface treatment on Au, the comparable hydrophobicity of MoS_2_ surface with that of Au surface suggests that MoS_2_ sensing surface in this study can be functionalized with specific antibody in a non-specific manner.

As the first step towards the underlying concept of real-time, electrical direct detection of charged biological species without a specific surface treatment, we investigate the sensor response to adsorption variation on 2D MoS_2_ crystal as a function of the concentration of charged human Immunoglobulin G (IgG). IgG is the most abundant antibody isotype in human, which has isoelectric point (*pI*) of ~8.5 (see, Fig. S1 for a theoretical estimate of 8.1). Phosphate buffer solution (PBS, *pH* = 7.2) is spiked with 7 different concentrations of human IgG that is positively charged at the *pH* of our measurements. Fig. 2(b) shows the sensor response to the adsorption of 7 different concentrations of human IgG onto MoS_2_ surface. Characteristics of MoS_2_ transistors have been shown to be variable with biomolecule adsorption. Here, the on-current (I_on_) of the MoS_2_ transistor shows negligible change with respect to the human IgG concentration, but the off-current (I_off_) of the MoS_2_ transistor significantly changes with human IgG concentration. We will discuss the theoretical model of the sensor in the next section, but the results are easy to understand intuitively: Without a positively charged human IgG, negative gate voltage during off-state depletes electrons in the n-type MoS_2_ channel (low off-current, see Fig. 2(b) and Fig. S3). Since human IgG (p*I* ~ 8.4–8.5) is positively charged at the measurement condition (p*H* = 7.2), binding of human IgG to the MoS_2_ surface causes an increase in electrons during off-state. However, during on-state, positive gate voltage accumulates electrons in the n-type MoS_2_ channel (high on-current) even without human IgG. Hence, the impact of human IgG bound to MoS_2_ surface on the accumulation of electrons is insignificant. As concentration of human IgG increased from 10 pg/mL to 100 μg/mL, the sensor response (I_off_) also increased (Fig. 2c), indicating that the amount of the adsorbed human IgG on the MoS_2_ sensor surface is approximately proportional to the applied human IgG concentration. Fig. 2(d) and 2(e) show the output characteristics of the device without IgG and with IgG concentration of 100 μg/mL. Before IgG binding, the sensor shows saturation at high *V_ds_* due to channel-pinch off. However, due to positive charge of IgG, the curves become linear reflecting transition from saturation to linear regime. These results provide a potentially important implication that 2D layered MoS_2_ can be an attractive candidate for a highly sensitive quantitative detection of biomolecular targets without an additional surface treatment.

We next investigate the dependence of sensor response on the applied PSA concentration in an anti-PSA functionalized 2D MoS_2_ nanosheet transistor as shown in Fig. 1(a). Fig. 3(a) shows the increase of off-state current after anti-PSA (antibody) with a concentration of 100 μg/mL is immobilized to the overall MoS_2_ device surface. The extreme increase of “off-current” in the transistor with anti-PSA physisorbed on the MoS_2_ nanosheet surface shows a similar trend from adsorption of human IgG onto MoS_2_ sensor surface, which is consistent with binding of positively charged biomolecules. Then, the off-state current decreases as PSA is selectively bound to the immobilized antibody on the MoS_2_ surface (Fig. 3(b)). Here, the variation of “off-current” due to the specific binding of negatively charged PSA with the antibodies allows us to monitor highly sensitive detection of PSA markers from 1 pg/mL to 10 ng/mL, and to compute quantitative bioassay from the binding of a charged biological species. As concentration of PSA increased from 1 pg/mL to 10 ng/mL, the increased sensing response with PSA concentration indicates that the amount of the adsorbed PSA on the anti-PSA immobilized MoS_2_ sensor surface is proportional to the PSA concentration. The minimum detectable concentration of PSA (see, Fig 3(b)) is 1 pg/mL, which is three orders of magnitude below the clinical cut-off level of 4 ng/mL. We will further suggest other approaches to improve sensitivity later in the paper. Fig. 3(c) and (d) shows the output characteristics of the transistor before and after PSA binding. Due to negative charge of PSA, the effective gate voltage on top of MoS_2_ surface decreases and this leads to current saturation (due to pinch-off) at high drain biases. While the sensor is highly sensitive, the selectivity of the sensor maybe degraded due to physisorption of parasitic molecules on the MoS_2_layer^27^. The selectivity of the sensor can however be improved by several methods such as prefilteration steps^28^, by increasing the incubation time for the adsorption of IgG probe molecules^27^ or by utilizing the competitive binding of proteins on a hydrophobic surface^29^,^30^.

The theory of classical FET-biosensors is well established, but its generalization to double-gated configuration^31^, especially in the presence of interface defects and pH dependent biomolecule charge require a careful analysis. A semiclassical approach is appropriate because the carrier transport in a sensor is dominated by scattering that can be addressed adequately by a drift-diffusion formulation.

Specifically, in order to interpret the experimental results and to develop a model for MoS_2_ biosensors, we solved for two-dimensional Poisson and continuity equation self-consistently throughout the device. Table 1 shows the numerical model used for determination of device characteristics (see, Fig. 4). Here, *ϕ* is the electrostatic potential, *n* and *p* are electron and hole concentrations in MoS_2_ layer, respectively; the channel is presumed n-doped with intrinsic doping density *N_d_* ~ 1 × 10^16^ cm^−3^, and *μ* and *D* are mobility and diffusion coefficients, respectively. For simplicity, the biomolecule charge is considered as a uniform surface charge sheet with density (*σ*_bio_) at the top MoS_2_ surface. This charge is obtained by calibrating the off-current values from the experiment with the simulation, see Fig. S2 (a). The response due to the biomolecules is compromised by the interface traps at top MoS_2_ surface (*σ*_it,top_) and MoS_2_/oxide interface(*σ*_it,bottom_). The parameters for protein charge and the device parameters are summarized in Supplementary Tables S3–S4, and the list of the symbols is described in Supplementary Table S5 and Supplementary Table S6.

Fig. 5(a) shows the transfer characteristics of the device (as a function of backgate voltage) for different PSA concentrations. The simulation results explain consistently the three key features observed in the experiments (refer, Fig. 3) *i.e.*, a) Saturation of current at large negative biases, b) the decrease in subthreshold-swing, and c) the decrease in on-current with the increase (not shown) in PSA concentration. The off-current results from the formation of a conduction channel (accumulated electrons) at the top MoS_2_ surface in response to the positive charge (at pH = 7.78) of the anti-PSA (antibody) at the top MoS_2_ surface. For the energy band-diagram along the channel, see. Fig. S3. The negatively charged PSA neutralizes some of the positive charge, and hence off-current decreases with increase in PSA concentration, see Fig. 5(b).

Similarly, the average subthreshold-swing (SS) of the device increases upon addition of positively charged anti-PSA due to increase in off-current. However, as the concentration of the negatively charged PSA is increased from 1 pg/mL to 10 ng/ml, the off-current decreases tenfold from ~2 nA to 0.2 nA (Fig. 5b vs. Fig. 3b), hence the average subthreshold slope reduces from 4.3 V/dec to 3.1 V/dec as shown in Fig. 5(c). Fig. 5(d) shows the I_ds_-V_ds_ characteristics for the device with anti-PSA, and with 1 ng/mL PSA concentration. The on-current of the device reduces as PSA is added to the solution, consistent with observations in Fig. 3.

Fig. 6 shows the comparison of sensitivity based on 4 different device parameters, *i.e.*, off-current, I_off_ (at V_gs_ = −40 V), average subthreshold-swing, SS (between −20 V and −10 V), linear threshold-voltage, V_T_ and on-current, I_on_ (V_gs_ = 16 V, V_ds_ = 10 V). And the figure explains why the off-current – more so than any other metric – is such a robust indicator of the capture of biomolecules.

Since the MoS_2_ sensor is accumulation mode device, the relative change in on-current of the transistor is very small. This is because the channel is highly conducting when the device is turned on and a small change in surface charge due to PSA binding causes a corresponding small change in the drain current. However, when the device is completely off, the channel is off and a small change in surface charge due to PSA binding brings relatively larger change in drain current. Unlike Lee^7^, the capture of PSA does not passivate/create any interface defects, therefore the very small change in V_T_ and S*S* reflect, only as a secondary metric, the changes in the off current.

The theoretical interpretation of the experimental results suggests opportunity for future optimization. The relatively large subthreshold slope of the MoS_2_ discussed in this paper (~2.2 V/dec before and ~4.3 V/dec after anti-PSA decorates the surface) reflects relatively high density of interface traps at the top MoS_2_ surface and MoS_2_/oxide interfaces. Fig. 7 suggests that the performance of MoS_2_ biosensor would improve approximately 40 times for P_PSA_ = 10 ng/mL, if surface treatment could reduce the defect density at the top MoS_2_ surface by a factor of 5, i.e., from 2 × 10^11^ # cm^−2^ to 4 × 10^10^ # cm^−2^. The reduction of defect density at the MoS_2_/oxide interface has even more dramatic consequences: Sensitivity at the same concentration (*ρ* = 10 ng/mL) improves by almost 4 orders of magnitude as the trap density is reduced from 8 × 10^11^ eV^−1^cm^−2^ to 1 × 10^10^ eV^−1^cm^−2^. Therefore, while the experimental results already demonstrate considerable potential of MoS_2_ based technology in terms of sensitivity, selectivity, fluid stability, and integration on Si-substrate, significant additional improvements are expected with improvement in surface treatment and interface passivation.

In summary, we report a comprehensive investigation on the highly sensitive biosensor platform based on multilayer MoS_2_ FETs to detect PSA. Our results demonstrate the successful use of MoS_2_ FET sensor in back-gated scheme without the need of the insulating oxide on the top of channel. The highly hydrophobic nature of the MoS_2_ surface allows it to serve the dual roles of the transducer and the recognition layer, with considerable improvement in sensitivity and significant simplification of device design. The absence of the oxide layer avoids the additional complexity involved in chemical treatment of the surface, and hence ensures effective coupling of biomolecule charge to the channel. Our theoretical model explains the experimental results consistently and indicates that the sensitivity can be further improved through surface treatment and interface passivation. Combined with the rapid advances in large-area synthesis methods of MoS_2_ such as CVD, these results deliver a compelling case of potentially using multilayer MoS_2_ FETs as biosensors.

## Author Contributions

J.L. and S.K. designed the experiments. Y.L. and W.C. fabricated the devices, and Y.L., H.P. and W.C. characterized the sensors. P.D. and M.A.A. planned the theoretical analysis; P.D. did the numerical simulation. J.L., S.K., M.A.A. and P.D. wrote the manuscript. All authors reviewed the manuscript. J.L., P.D. and Y.L. contributed equally to this work.

## Supplementary Material

Supplementary Information Supplementary Information

## Figures and Tables

**Figure 1:**
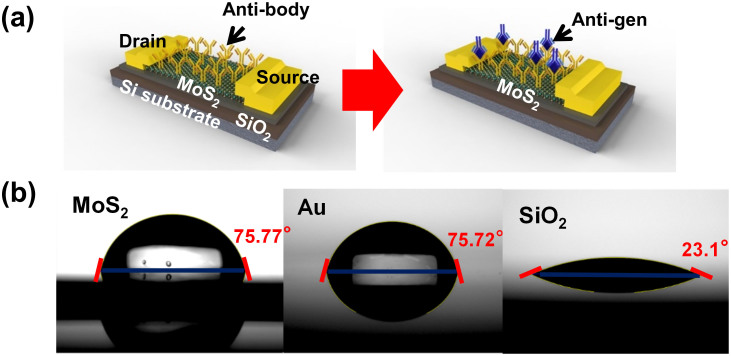
A MoS_2_ nanosheet biosensor and contact angles of different surfaces. (a) Schematic of a MoS_2_ biosensor configured as a PSA, detecting label-free immunoassay, illustrating PSA antibody functionalized MoS_2_ surface (top) and subsequent binding of PSA antigen with antibody receptors. The MoS_2_ nanosheet biosensor consists of a gate insulator of SiO_2_ (300 nm) and a drain-source metal contact of Ti/Au (15 nm/300 nm) (b) The water contact angle measurement to confirm hydrophobic characteristics of different substrates: the water contact angle of MoS_2_, Au, and SiO_2_ substrate are 75.75°, 75.72°, and 23.1°, respectively. The contact angle of MoS_2_ surface, which is more hydrophobic than Si-based substrates, is comparable to that of Au surface. This suggests that MoS_2_ nanosheet is an excellent candidate for functionalizing antibody and protein due to its hydrophobic surface.

**Figure 2:**
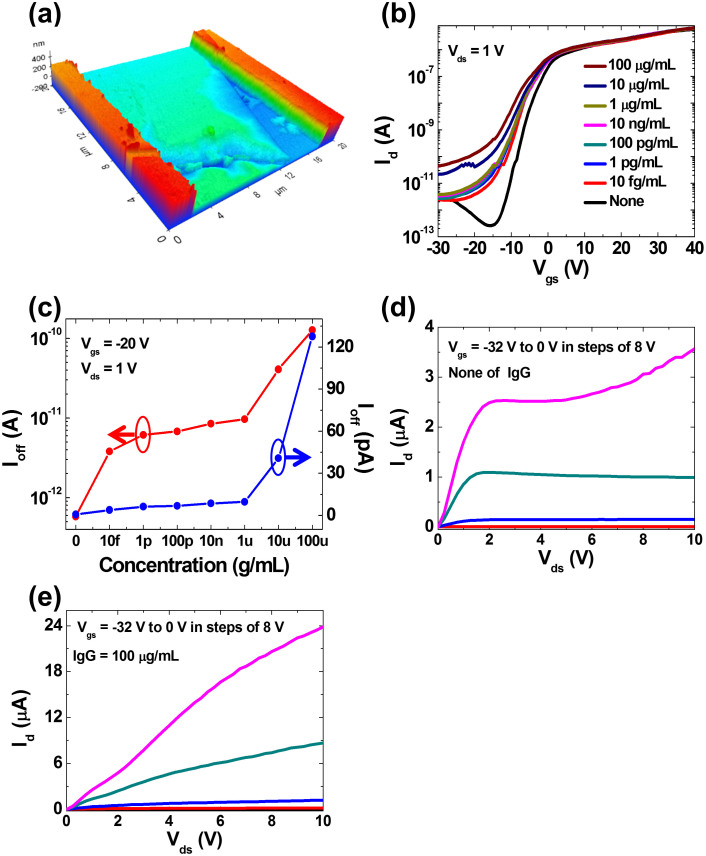
Adsorption of human IgG onto MoS_2_ sensor surface. (a) 3D AFM topography of multi-layer MoS_2_ with thickness of ~80 nm. (b) Transfer characteristics under various concentration of the human IgG from 0 to 100 μg/mL at V_ds_ = 1 V. (c) Plots of off-current versus human IgG concentration show an increase of off-current with increasing concentration of the human-IgG and abrupt increase of off-current at specific concentration of 10 fg/mL for V_gs_ = −20 V and V_ds_ = 1 V. Arrows indicate appropriate axis (red: log-scale, blue: linear-scale). (d), (e) Output characteristics under human IgG conditions of 0 and 100 μg/mL from V_gs_ = −32 V to V_gs_ = 0 V in steps of 8 V, respectively. Following adsorption of human IgG on MoS_2_ surface, the drain current exhibits 6-fold increase at a high drain voltage and saturation currents disappear due to the immobile charge of human IgG on the MoS_2_ nanosheet.

**Figure 3:**
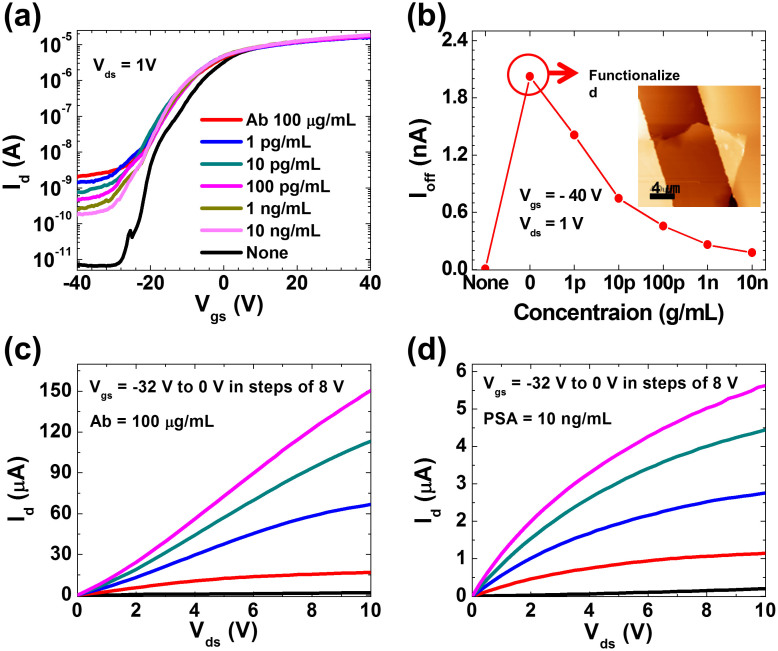
MoS_2_ nanosheet biosensor for PSA detection. (a) Transfer characteristics of MoS_2_ transistor biosensor functionalized by anti-PSA of 100 μg/mL under various PSA concentrations. The surface of MoS_2_ nanosheet is initially functionalized with anti-PSA antibody receptor. The binding of charged anti-PSA receptors to MoS_2_ nansosheet gives rise to the off-current increase from 10^−12^ to 10^−9^ A of a transistor. (b) Change of the off-current versus various PSA concentrations for an anti-PSA modified n-type MoS_2_ transistor at the condition of V_gs_ = −40 V and V_ds_ = 1 V. As the concentration of PSA increases from 1 pg/mL to 10 ng/mL, the amount of selective binding of PSA to the anti-PSA immobilized MoS_2_ nanosheet is approximately proportional to the PSA concentration. Inset shows an AFM image of MoS_2_ device with thickness of ~70 nm, width of 12.48 μm and length of 11.64 μm. (c), (d) Output characteristics of MoS_2_ nanosheet biosensor with functionalized anti-PSA concentration of 100 μg/mL and PSA concentration of 10 ng/mL from V_gs_ = −32 V to V_gs_ = 0 V in steps of 8 V, respectively. Current saturation appears and the current decreases by 10-fold due to the charge neutralization associated with the selective reaction between PSA antibody and PSA antigen on the MoS_2_ nanosheet.

**Figure 4:**
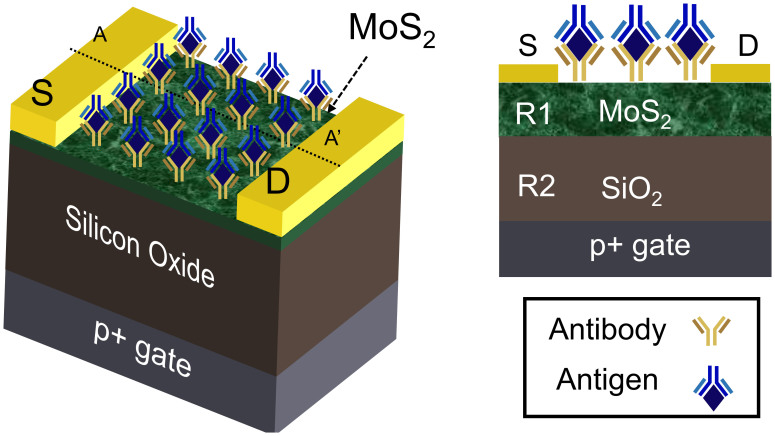
Schematic of the device used for numerical simulation of device characteristics.

**Figure 5:**
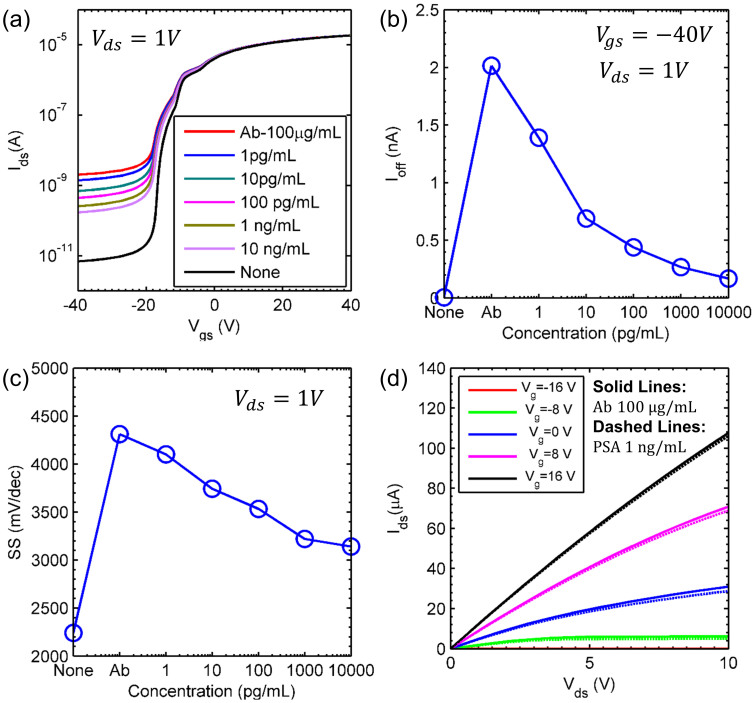
Simulated MoS_2_ device characteristics for PSA detection. (a) Transfer characteristics at *V_ds_* = 1 *V*; (b) Off-current at *V_ds_* = 1 *V* and *V_gs_* = −40 *V* as a function of PSA concentration; (c) Average Subthreshold swing (between −10 *V* and −20 *V*) as a function of the PSA concentrations; and (d) Output characteristics of MoS_2_ sensor with and without PSA on a surface, which has been pre-functionalized with anti-PSA.On-current decreases due to negative charge of PSA.

**Figure 6:**
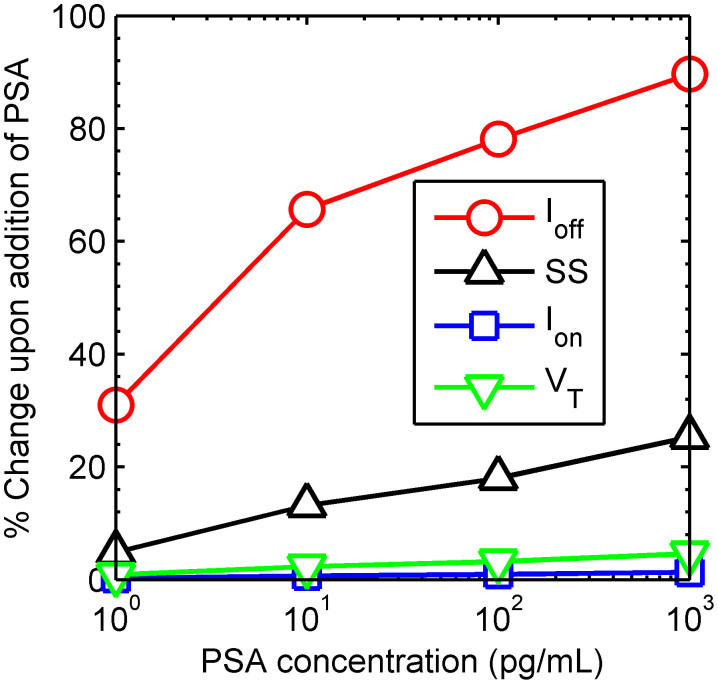
Comparison of sensitivity based on 4 different device parameters. The base value used is for a MoS_2_ surface with anti-PSA bound on its surface. The off-current shows a considerably larger change upon PSA binding as compared to the subthreshold-swing, threshold voltage and on-current.

**Figure 7:**
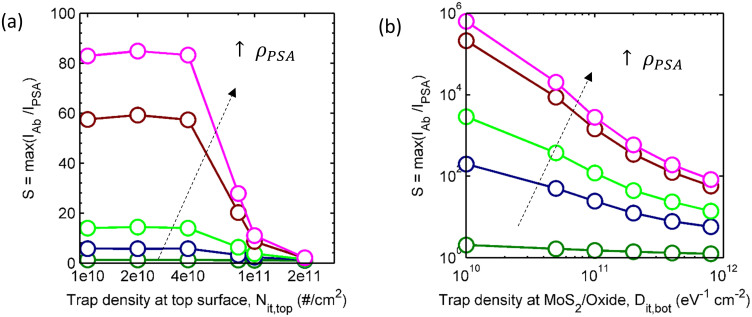
Improving the sensitivity by interface passivation. (a) Variation of MoS_2_ sensitivity as a function of interface trap densities at top surface of MoS_2_ for different PSA concentrations (with *D_it,bot_* = 8 × 10^11^ eV^−1^cm^−2^)) b) Variation of MoS_2_ sensitivity as a function of interface trap density at MoS_2_-oxide interface (with *N_it,top_* = 4 × 10^10^ eV^−1^cm^−2^) for different PSA concentrations: 1 pg/mL (Green), 10 pg/mL (Blue), 100 pg/mL (Lime), 1 ng/mL (Brown), 10 ng/mL (Magenta).

**Table 1:** Numerical equations for MoS_2_ sensor

Regions 1 & 2 (R1 and R2)	−∇.(*ε*∇*ϕ*) = *q*(*p* – *n* + 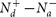 )
Region 1 (R1)	∇.(*qD_n_*∇*n* – *qμ_n_n*∇*ϕ*) = 0
	∇.(−*qD_p_*∇*p* – *qμ_p_p*∇*ϕ*) = 0
Source contact	*ϕ* * = * 0
Drain contact	*ϕ* * = * *V_ds_*
Gate contact	*ϕ* * = * *V_gs_* – *ϕ_ms_*
	where
	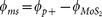
MoS_2_ – SiO_2_ interface	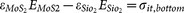
Top MoS_2_ surface