# Supplementary material for: Two-dimensional Layered MoS2 Biosensors Enable Highly Sensitive Detection of Biomolecules
Source: Sci Rep. 2014 Dec 17;4:7352. doi: 10.1038/srep07352 (PMC4268637; doi:10.1038/srep07352)
Supplement: Supplementary Information [file srep07352-s1.pdf]

Supporting Material

Two-dimensional Layered MoS_2 Biosensors enable Highly Sensitive Detection of Biomolecules

Joonhyung Lee[†,1], Piyush Dak[†,*,2], Yeonsung Lee[†,1], Heekyeong Park[1], Woong Choi[3], Muhammad A. Alam[*,2], and Sunkook Kim[*,1]

[1]*Multi-Functional Nano/Bio Electronics Lab., Department of Electronics and Radio Engineering, Kyung Hee University, Gyeonggi, 446-701, South Korea*
[2]School of Electrical and Computer Engineering, Purdue University, West Lafayette, IN 47907, USA
[3]*School of Advanced Materials Engineering, Kookmin University, Seoul 136-702, Korea*

[†]These authors contributed equally to this publication
*e-mail: alam@purdue.edu; seonkuk@khu.ac.kr; pdak@purdue.edu

1. Electrical Measurement of MoS_2 biosensor

a) Adsorption of Human IgG onto MoS_2 sensor surface: The MoS_2 surface was incubated with human IgG (in phosphate buffer saline (PBS), pH 7.2) for 15 minutes, followed by rinsing with PBS for 1 minute and drying under a nitrogen gas. Then 3 Au-probe tips using the conventional manipulator were connected to the gate, drain, and source electrode in MoS_2 transistor. Electrical characterizations were carried out with current-voltage measurements (Keithley, Semiconductor Characterization System 4200-SCS). In this regard, seven different concentration of human IgGs (10 pg/mL ~ 10 μg/mL) were analyzed sequentially to measure the on/off currents for a transistor.

b) Label-free immunoassay: Anti-PSA (100 μg/mL in PBS, pH 7.2) was immobilized on the MoS_2 surface for 1 hour. In order to avoid evaporation, the dispensing process was performed inside a humidity chamber. The chip was rinsed with PBS buffer for 1 minute. Electrical

characterizations were carried out with current-voltage measurements (Keithley, Semiconductor Characterization System 4200-SCS). The anti-PSA functionalized MoS_2 surface was reacting with PSA (in phosphate buffer saline (PBS), pH 7.78) for 10 minutes, followed by rinsing with PBS (pH 7.78) for 1 minute, where six different concentrations of PSA (1 pg/mL ~ 100 ng/mL in PBS, pH 7.78) were analyzed sequentially to measure the electrical curves of MoS_2 biosensor.

2. Determination of Human IgG, anti-PSA and PSA charge

The protein sequence for different segments of Human IgG and anti-PSA was obtained from various sources. The protein sequence for PSA was obtained from protein databank. Supplementary Table 1 tabulates the sequence for protein segments and corresponding reference.

**Supplementary Table S1: Determination of protein sequence**		
**Segment**	**FASTA Sequence**	**Ref**
**Human IgG**		
Fab Light Chain (A)	SDIQMTQSPSSLSASVGDRVTITCRASQSVSSAVAWYQQKPGKAPKLLIYSASSLYSGVPS<br>RFSGSRSGTDFTLTISSLQPEDFATYYCQQYSSYSSLFTFGQGTKVEIKRTVAAPSVFIFP<br>PSDEQLKSGTASVVCLLNNFYPREAKVQWKVDNALQSGNSQESVTEQDSKDSTYSLSSTLT<br>LSKADYEKHKVYACEVTHQGLSSPVTKSFNRGEC	PDB [1]
Fab Heavy Chain (B)	EISEVQLVESGGGLVQPGGSLRLSCAASGFNVKTGLIHWVRQAPGKGLEWVAYISPYYGST<br>SYADSVKGRFTISADTSKNTAYLQMNSLRAEDTAVYYCAREYYRWYTAIDYWGQGTLVTVS<br>SASTKGPSVFPLAPSSKSTSGGTAALGCLVKDYFPEPVTVSWNSGALTSGVHTFPAVLQSS<br>GLYSLSSVVTVPSSSLGTQTYICNVNHKPSNTKVDKKVEPKSCDKTH	PDB [1]
Fc Heavy Chain (Hinge, CH_2, CH_3 region)	EPKSCDKTHTCPPCPAPELLGGPSVFLFPPKPKDTLMISRTPEVTCVVVDVSHEDPEVKFN<br>WYVDGVEVHNAKTKPREEQYNSTYRVVSVLTVLHQDWLNGKEYKCKVSNKALPAPIEKTIS<br>KAKGQPREPQVYTLPPSRDELTKNQVSLTCLVKGFYPSDIAVEWESNGQPENNYKTTPPVL<br>DSDGSFFLYSKLTVDKSRWQQGNVFSCSVMHEALHNHYTQKSLSLSPGK	UniProt [2]

**anti-PSA (IgG_{2a})**		
Chain C region	AKTTAPSVYPLAPVCGDTTGSSVTLGCLVKGYFPEPVTLTWNSGSLSSGVHTFPAVLQSDL YTLSSSVTVTSSTWPSQSITCNVAHPASSTKVDKKIEPRGPTIKPCPPCKCPAPNLLGGPS VFIFPPKIKDVLMISLSPIVTCVVVDVSEDDPDVQISWFVNNVEVHTAQTQTHREDYNSTL RVVSALPIQHQDWMSGKEFKCKVNNKDLPAPIERTISKPKGSVRAPQVYVLPPPEEEMTKK QVTLTCMVTDFMPEDIYVEWTNNGKTELNYKNTEPVLDSDGSYFMYSKLRVEKKNWVERNS YSCSVVHEGLHNHHTTKSFSRTPGK	UniProt [2]
Heavy chain V region	QVQLQQPGAELVRPGSSVKLSCKASGYTFTSYWMDWVKQRPGQGLEWIGNIYPSDSETHYN QKFKDKATLTVDKSSSTAYMQLSSLTSEDSAVYYCAR	UniProt [2]
Light Chain	DVVMTQSPKTISVTIGQPASISCKSSQRLLNSNGKTFLNWLLQRPGQSPKRLIYLGTKLDS GVPDRFTGSGSGTDFTLKISRVEAEDLGVYYCWQGTHFPYTFGGGTKLEIKRADAAPTVSI FPPSSEQLTSGGASVVCFLNNFYPKDINVKWKIDGSERQNGVLNSWTDQDSKDSTYSMSST LTLTKDEYERHNSYTCEATHKTSTSPIVKSFNRNEC	PDB [1]
**PSA**		
**Complete Sequence**	IVGGWECEKHSQPWQVLVASRGRAVCGGVLVHPQWVLTAAHCIRNKSVILLGRHSLFHPED TGQVFQVSHSFPHPLYDMSLLKNRFLRPGDDSSHDLMLLRLSEPAELTDAVKVMDLPTQEP ALGTTCYASGWGSIEPEEFLTPKKLQCVDLHVISNDVCAQVHPQKVTKFMLCAGRWTGGKS TCSGDSGGPLVCNGVLQGITSWGSEPCALPERPSLYTKVVHYRKWIKDTIVANP	PDB [1]

In order to determine the protein charge at a given pH, we determined the charge on each amino acid for a given pH and summed it over the complete sequence of protein. Fig. S1 shows the plot of protein charge (Human IgG, anti-PSA, and PSA) as a function of pH. Once the charge on a protein (Q_{protein}) at a particular pH is known, the surface charge is given by $\sigma_s = q_{\text{protein}} N_s$ where N_s is the density of protein molecules on the surface. Supplementary Table S2 & S3 show the equation used for estimation of the protein charge and count of number of charged amino acids in protein molecule, respectively.

**Supplementary Table S2: Calculation of Protein Charge**	
Fraction of base with $pK = -log10[K_{bi}]$ in ionized form	$f_{bi} = \frac{[H^+]}{[H^+] + K_{bi}}$
Fraction of acid with $pK = -log10[K_{ai}]$ in ionized form	$f_{ai} = \frac{K_{ai}}{[H^+] + K_{ai}}$

Charge $\left(q_{protein}\right)$ on protein molecule	$$Q = q\left(\sum_i i\, f_{bi} - \sum_j j\, f_{aj}\right)$$ where i is the number of bases in the protein sequence and j is the number of acids in the protein sequence

Supplementary Table S3: Number of charged amino acids in human IgG, anti-PSA and PSA and their corresponding pK [3]

Amino Acid	pK	Human IgG	anti-PSA	PSA
**K (Base) Lysine**	10	94	92	12
**R (Base) Arginine**	12	38	40	10
**H (Base) Histidine**	6.5	26	26	11
**D (Acid) Aspartate**	4.4	54	64	11
**E (Acid) Glutamate**	4.4	66	60	11
**C (Acid) Cysteine**	8.5	34	34	10
**Y (Acid) Tyrosine**	10	68	50	4
**NH_2 Terminal Group**	8	1	1	1
**COOH Terminal Group**	3.1	1	1	1

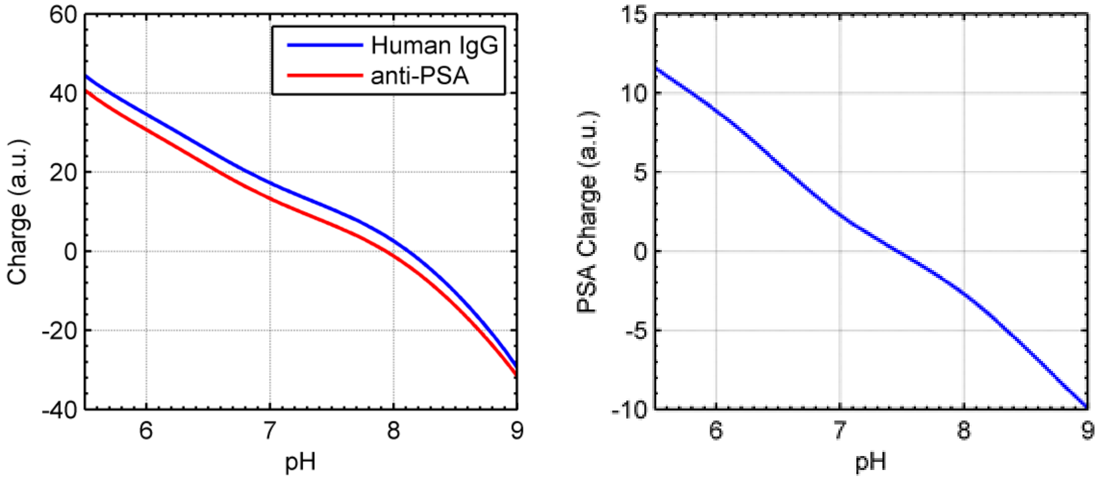

Fig. S1 (a) Human IgG, anti-PSA, and (b) PSA charge as a function of pH. The isoelectric point (IP) of human IgG, anti-PSA and PSA are 8.12, 7.94 and 7.46, respectively.

3. Calibration of Surface Densities of Biomolecules

The effective surface densities (after accounting for ionic screening) of anti-PSA ($\mathrm{N_{anti-PSA}}$) and PSA ($\mathrm{N_{PSA}}$) on MoS_2 surface are obtained by calibrating the experimental off-value current values with the theoretical estimates (refer, Fig. S2(a)). Briefly, the charge per anti-PSA molecule at pH=7.78 is $\mathrm{q_{anti-PSA}(pH = 7.78) = 2.68}\,q$ and the charge per PSA molecule at pH=7.78 is $\mathrm{q_{PSA}(pH = 7.78) = -1.52}\,q$, where q is electronic charge. The net charge density on MoS_2 surface for a given pH is given by, $\mathrm{Q_{surface}(pH) = q_{anti-PSA}N_{anti-PSA} + q_{PSA}N_{PSA}}$. Once the surface charge density is known by calibration with the experimental data, we can find the effective surface density of PSA at different bulk PSA concentrations. Interestingly, the surface charge density of PSA follows a logarithmic trend with respect to its bulk concentration, ρ *i.e.* $\mathrm{Q_{PSA} = q_{PSA}N_{PSA} = q_{PSA}N_{IgG}}\,\zeta\, log\left(\frac{\rho}{\rho_0} + 1\right)$ where ζ and ρ_0 are constants. Fig. S2 (b) shows the fraction of effective PSA concentration binded to the anti-PSA (antibodies) as a function of bulk PSA concentration. The symbols

represent the values obtained by fitting the experimental data and line represents the fit to the expression.

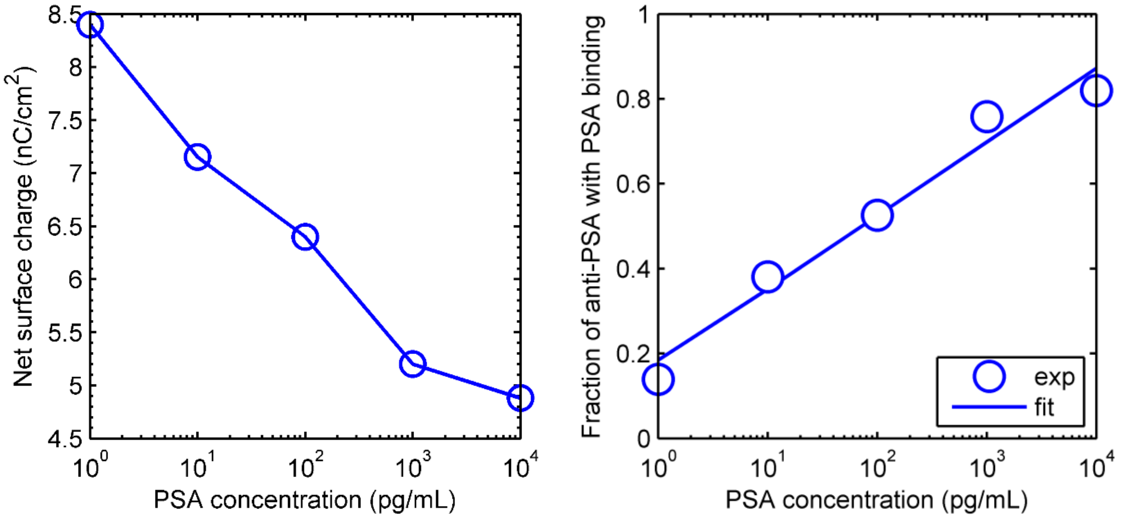

Fig. S2 (a) Calibration curve for surface charge density due to anti-PSA/PSA binding as a function of PSA bulk concentration. (b) The fraction of anti-PSA molecules bound to PSA molecules as a function of PSA concentration. Symbols are values obtained from (a) and fit corresponds to expression $f \sim \zeta\, log\left(\frac{\rho}{\rho_0}+1\right)$.

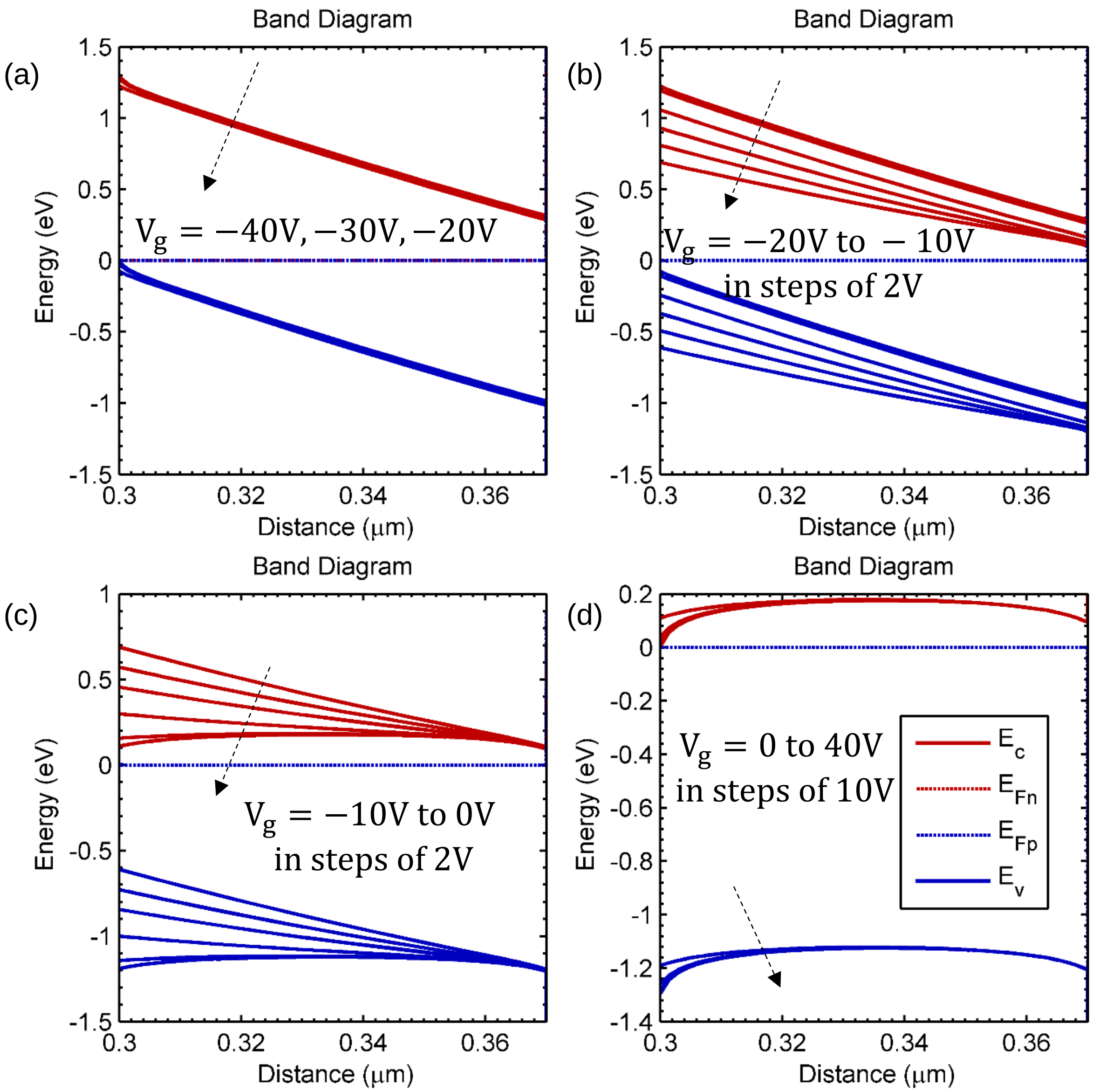

Fig. S3 Band diagram of the MoS_2 channel in different operation regimes (a) Off-current regime (b) –(c) Subthreshold regime (d) On-current regime

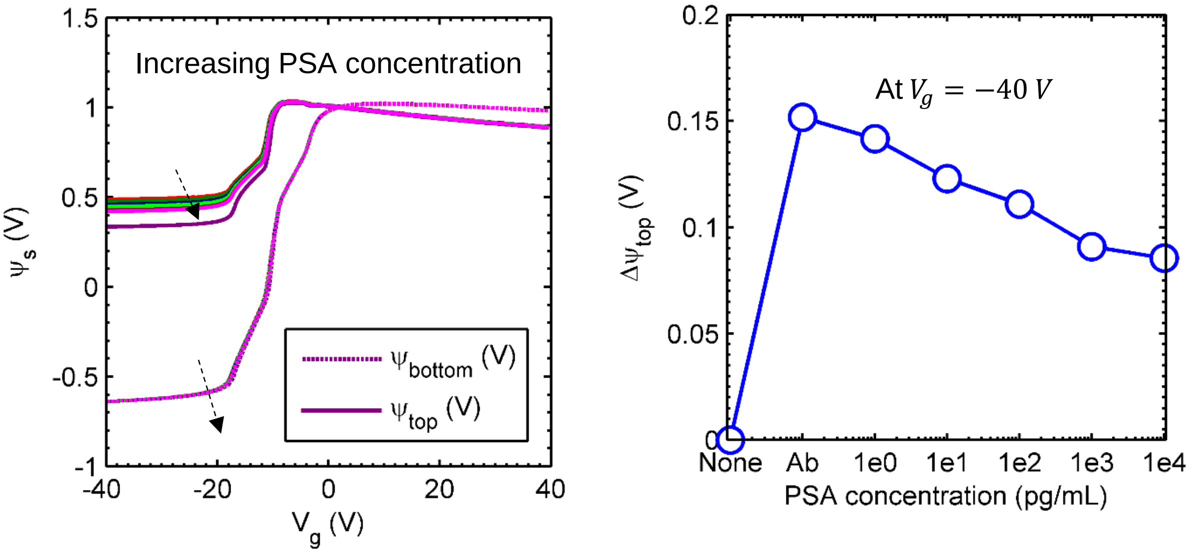

Fig. S4 (a) The Variation of surface potential at top (ψ_{top}) and bottom MoS_2 surfaces (ψ_{bottom}) as a function of gate bias for different biomolecule concentrations at $V_{ds} = 1V$. The MoS_2-oxide interface potential is hardly affected by the biomolecule concentration. Color code: No PSA/IgG (Purple), After IgG functionalization (red), With PSA concentrations: $1\ \mathrm{pg/ml}$ (Green), $10\ \mathrm{pg/ml}$ (Blue), $100\ \mathrm{pg/ml}$ (Lime), $1\ \mathrm{ng/ml}$ (Maroon), $10\ \mathrm{ng/ml}$ (Magenta) (b). Variation of MoS_2 top surface potential for $V_g = -40\ V$ as a function of biomolecule concentration.

**Supplementary Table S4: Simulation parameters**		
**Parameter**	**Value**	**Reference**
Multilayer MoS_2 band gap	1.3 eV	4
Multilayer MoS_2 electron affinity	4.3 eV	4
Intrinsic doping density	$1 \times 10^{16}\ \mathrm{cm}^{-3}$	4
Electron/Hole mobility	$50\ \mathrm{cm}^2/\mathrm{Vs}$	Calibrated
Workfunction of $p+$ substrate	5.17 eV	Calculated
Fixed-charge density at oxide-MoS_2 interface	$2.6 \times 10^{11}\ \mathrm{cm}^{-2}$	Calibrated
Uniform donor density at oxide-MoS_2 interface	$8 \times 10^{11} \mathrm{eV}^{-1}\mathrm{cm}^{-2}$	Calibrated
Fixed-charge density at MoS_2 – electrolyte interface	$2.8 \times 10^{11}\ \mathrm{cm}^{-2}$	Calibrated
Series Resistance	20 KΩ	Calibrated

**Supplementary Table S5: List of Symbols for charge transport in MoS_2**	
**Parameter Name**	**Symbol**
Electrostatic Potential at any point in the device	ϕ
Electron concentration	n
Hole concentration	p
Electron mobility	μ_n

Hole mobility	μ_p
Electron diffusion coefficient	D_n
Hole diffusion coefficient	D_p
Trap density at MoS_2-electrolyte interface	$\sigma_{\mathrm{it,top}}$
Trap density at MoS_2-oxide interface	$\sigma_{\mathrm{it,bottom}}$
Work function of p+ substrate	ϕ_{p+}
Work function of MoS_2	ϕ_{MoS_2}
Intrinsic doping density in MoS_2	N_d
Drain to source voltage	V_{ds}
Gate to source voltage	V_{gs}
Permittivity in MoS_2	ε_{MoS_2}
Permittivity in SiO_2	ε_{SiO_2}
Electronic Charge	q

Supplementary Table S6: List of symbols for protein charge calculation

**Parameter**	**Symbol**
Hydronium ion concentration in the buffer solution	$[\mathrm{H}^+]$
Dissociation constant of jth acidic group in protein	K_{aj}
Association constant of ith base in protein	K_{bi}
Fraction of ionized j^{th} acidic group in protein sequence	f_{aj}
Fraction of ionized i^{th} basic group in protein sequence	f_{bi}
Charge per protein molecule	q_{bio}

References:

1. Protein Databank, http://www.rcsb.org/pdb/home/home.do
2. UniProt, http://www.uniprot.org/
3. DTASelect, http://isoelectric.ovh.org/files/isoelectric-point-theory.html
4. Kim, S. *et al.* High-mobility and low-power thin-film transistors based on multilayer MoS2 crystals. *Nat. Commun.* **3,** 1011 (2012).
